# Serum neurofilament light and white matter characteristics in the general population: a longitudinal analysis

**DOI:** 10.1007/s11357-023-00846-x

**Published:** 2023-06-07

**Authors:** Marco Hermesdorf, Niklas Wulms, Aleksandra Maceski, David Leppert, Pascal Benkert, Heinz Wiendl, Jens Kuhle, Klaus Berger

**Affiliations:** 1 Institute of Epidemiology and Social Medicine, University of Münster, Münster, Germany; 2 Multiple Sclerosis Centre, Neurology, Departments of Head, Spine and Neuromedicine, Biomedicine and Clinical Research, University Hospital Basel and University of Basel, Basel, Switzerland; 3 Clinical Trial Unit, Department of Clinical Research, University Hospital Basel and University of Basel, Basel, Switzerland; 4 Department of Neurology With Institute of Translational Neurology, University Hospital Münster, Münster, Germany

**Keywords:** Neurofilament light, Age, Aging, Diffusion tensor imaging, Fractional anisotropy, White matter lesions

## Abstract

Neurofilament light polypeptide (NfL) is a component of the neuronal cytoskeleton and particularly abundant in large-caliber axons. When axonal injury occurs, NfL is released and reaches the cerebrospinal fluid and the blood. Associations between NfL and white matter alterations have previously been observed in studies based on patients with neurological diseases. The current study aimed to explore the relationship between serum NfL (sNfL) and white matter characteristics in a population-based sample. The cross-sectional associations between sNfL as dependent variable, fractional anisotropy (FA), and white matter lesion (WML) volume were analyzed with linear regression models in 307 community-dwelling adults aged between 35 and 65 years. These analyses were repeated with additional adjustment for the potential confounders age, sex, and body mass index (BMI). Longitudinal associations over a mean follow-up of 5.39 years were analyzed with linear mixed models. The unadjusted cross-sectional models yielded significant associations between sNfL, WML volume, and FA, respectively. However, after the adjustment for confounders, these associations did not reach significance. In the longitudinal analyses, the findings corroborated the baseline findings showing no significant associations between sNfL and white matter macrostructure and microstructure beyond the effects of age. In synopsis with previous studies in patients with acute neurological diseases showing a significant association of sNfL with white matter changes beyond the effects of age, the present results based on a sample from the general population suggest the perspective that changes in sNfL reflect age-related effects that also manifest in altered white matter macrostructure and microstructure.

## Introduction

Neurofilament light polypeptide (NfL) is a component of the neuronal cytoskeleton and particularly abundant in large-caliber axons [1] where it contributes to radial axonal growth and axonal stability [2, 3]. In case of loss of axonal neurofilaments, growth of the axon diameter is constrained and consequentially leads to a slower propagation of nerve impulses along the axon [4]. When axonal injury occurs, e.g., in amyotrophic lateral sclerosis, multiple sclerosis, or traumatic brain injury, the axon subsequently disintegrates into smaller fragments, leading to a release of NfL into the extracellular fluid [5]. From there, NfL also reaches the cerebrospinal fluid and the blood [6, 7].

NfL has been proposed as a specific biomarker for neuro-axonal damage irrespective of cause and the monitoring of disease activity, particularly in the domain of neurodegenerative diseases [8, 9], traumatic brain injury [10], and cerebral small vessel disease [11], but also increases with age in healthy participants [12, 13]. With single molecular array (SIMOA) assays, it is possible to quantify NfL levels in the blood serum (sNfL) down to the picogram per milliliter range [14]. The increasing application of sNfL as a biomarker is less invasive compared to the assessment of NfL in cerebrospinal fluid and therefore more suitable as a potential future screening or monitoring tool for acute neuro-axonal damage. In patients with multiple sclerosis, it has been shown that sNfL is associated with white matter lesion (WML) volume [15] and decreased fractional anisotropy (FA) [16], a marker of white matter microstructural properties. Similar associations between WML, FA, and sNfL have been observed in patients with autosomal dominant Alzheimer’s disease [17]. Decreased FA related to higher plasma NfL levels has been reported in patients with frontotemporal dementia [18]. However, while damage to white matter is most often permanent, NfL levels peak after acute injury and decrease after a short period of time (typically 6–9 months) [19]. In particular, increased NfL levels have been observed in individuals 7–10 days after participating in a contact sports event before NfL concentrations subsequently normalized following a 3-month period [20]. Similar findings have been reported in stroke patients where sNfL peaked 3 weeks after the incident and returned to almost baseline levels after a period of 3–5 months [21]. It is particularly important to characterize sNfL-related changes in white matter characteristics across the lifespan in the general population to better distinguish non-diseased from diseased states and to improve the interpretability of sNfL. The purpose of the present study was to analyze cross-sectional and longitudinal associations of sNfL with macrostructural and microstructural white matter characteristics, operationalized as WML volume and FA, in a population-based sample of the general population over the course of several years.

## Methods

### Participants

The present analyses is based on the population-based control cohort from the longitudinal BiDirect Study conducted in Münster, Germany [22, 23]. Participants aged between 35 and 65 years were randomly drawn from the local population register and invited to the study center to take part in the extensive medical examination program which included magnetic resonance imaging, taking blood samples, a physical examination, a cognitive assessment, and a face-to-face interview. Baseline response rate was 41.5%, and participants were reinvited after approximately 4 years to again participate in the second follow-up examination of the BiDirect Study. The re-examination program followed the same standard operating procedures and used the same devices as the baseline assessment to ensure comparability across time points. Participants did not receive any financial or non-financial incentive to take part in the examination program.

For the present analyses, participants with available sNfL who underwent diffusion-weighted and fluid-attenuated inversion recovery magnetic resonance imaging and returned for the second follow-up examination were included (*n* = 350). Artifacts and insufficient image quality were the only exclusion criteria. Following quality screening of the diffusion-weighted images, 43 participants were excluded due to artifacts and insufficient image quality observed at least at one of the two time points, resulting in a final sample size of 307 participants. Written informed consent was obtained from all participants.

### Image acquisition and preprocessing

The MR images were collected on the same 3 Tesla scanner (Intera, Philips, Best, NL) in both examinations. Diffusion-weighted images were acquired along 20 non-collinear directions together with one image without diffusion weighting (*b* = 0 s/mm^2^) and the following parameters: repetition time (TR) = 5900 ms, echo time (TE) = 95 ms, 90° flip angle, 128 × 128 matrix, 240 × 240 mm^2^ field of view (FOV), 36 axial slices, 3.6-mm slice thickness, reconstructed to pixel size = 0.94 × 0.94 mm^2^, and *b* = 1000 s/mm^2^. The scans were preprocessed using FSL [24, 25] version 6.0.2. Eddy current distortions and head motion were corrected with FDT and skull stripped with BET. DTIFIT was used to fit diffusion tensors and generate FA maps. These were quality checked by visually inspecting 9 slices of each image with slicesdir. An in-house script (git.io/JE1Nj) was used for additional quality screening whereby several signal-to-noise ratios were computed, and images with prominent deviations were further inspected. In order to improve the alignment between the two time points and to minimize a potential bias towards one of the two time points, we followed a previously proposed approach [26]. In brief, FA maps from the second follow-up were linearly registered to the baseline FA maps and vice versa. The halfway point [27] between the FA maps from both time points was computed, and a subject-specific mean FA template was generated. The initial FA maps from both time points were then linearly registered to the respective subject-specific template. In the course of the subsequent tract-based spatial statistics (TBSS) procedure, the smoothed images were nonlinearly registered to the FMRIB58_FA template. A mean image of all FA maps was created and thinned to produce a white matter skeleton representing the tract centers across the sample. Individual FA maps from both timepoints were then projected onto this white matter skeleton, whereby a threshold of 0.2 was applied to the mean FA skeleton image. The averaged FA values across the white matter skeleton (“global FA”) were extracted for all participants and timepoints. Furthermore, FA values for major tracts were extracted using the JHU white matter tractography atlas [28].

Fluid-attenuated inversion recovery (FLAIR) images were collected with the following parameters: TR = 11,000 ms, TE = 120 ms, inversion time (TI) 2600 ms, 90° flip angle, 352 × 206 matrix, 230 × 186 mm^2^ FOV, 27 axial slices, 4-mm slice thickness, inter-slice gap = 1 mm, reconstructed to pixel size = 0.45 × 0.45 mm^2^, and dimensions 512 × 512 × 27. Three-dimensional T1-weighted (T1w) gradient echo sequence with inversion prepulse (TFE) was collected with the following parameters: TR = 7.26 ms, TE = 3.56 ms, TI 404 ms, 9° flip angle, 256 × 256 matrix, 160 sagittal slices, 2-mm slice thickness, reconstructed to 1-mm slice thickness by zero filling in *k-*space, and reconstructed to pixel size = 1 × 1 mm^2^.

A subset of 201 FLAIR scans was segmented manually for WML with FSLeyes version 0.22.1 [29]. Two raters segmented the images interchangeably after instruction and training. The T1w and FLAIR scans were reoriented, cropped, defaced, and then processed separately with the fsl_anat pipeline implemented in FSL version 6.0.3 [24, 25] The output of both pipelines resulted in bias-corrected and brain-extracted images. The T1w sequences were then realigned into FLAIR space using FLIRT (affine, 6 degrees of freedom). The brain-extracted, bias-corrected and realigned T1w, FLAIR images, and WML lesion masks were used for the training of the Brain Intensity AbNormality Classification Algorithm (BIANCA) [30]. The trained model was then used to predict WML masks for all subjects of the study. The predicted WML masks were thresholded with 0.8 to extract the whole-brain total WML volume in ml. The whole workflow was wrapped with one in-house built script [31].

### Serum neurofilament light chain measurements

Non-fasting blood samples were collected from each consenting participant immediately after the interview. Samples were processed directly in the study center within 2 h. After centrifugation, serum aliquots (370 and 500 μl) were prepared for long-term storage at – 80 °C. sNfL was quantified with a commercially available kit (NF-Light, Quanterix) that was applied on the single molecule array HD-X analyzer (Quanterix, Lexington, MA, USA), as previously described. Samples were run in duplicate by board-certified technicians blinded to clinical information. The coefficients of variation for all samples reported were < 15%.

### Data analyses

In a first step, linear regression models were fitted to the cross-sectional baseline data to separately analyze the associations between log_10_-transformed sNfL levels as dependent variable and global mean FA (model 1) as well as log_10_-transformed WML volume (model 2) as independent variables. These analyses were subsequently repeated with additional adjustment for age, sex, and BMI as potential confounders. In order to analyze potential tract-specific associations with NfL, we conducted linear regression models with the mean FA values for 12 major tracts as respective independent variables, adjusted for age, sex, and BMI.

Longitudinal associations between NfL and white matter characteristics were analyzed with linear mixed models. The log_10_ sNfL levels served as dependent variable whereas global mean FA (model 1) and WML volume (model 2) served as independent variables, respectively. The mixed models were adjusted for age, sex, and BMI. These variables were entered as fixed effects in addition to a random intercept in order to account for the correlated nature of repeated measurements.

We assumed that NfL releasing changes in the cytoskeleton, changed FA, and WML volume are potential reflections of the same underlying process and thus occurring in parallel. However, sNfL levels peak approximately 3 week after a neurological incident [21] while white matter changes can be detected within hours using magnetic resonance imaging [32, 33]. Therefore, we designated sNfL as the dependent variable in the current analyses. R version 4.03 [34] and RStudio version 1.4.1103 [35] were used for the statistical analyses. *P* values below 0.05 (two-tailed) were considered significant.

## Results

### Demographic characteristics

**Fig. 1:**
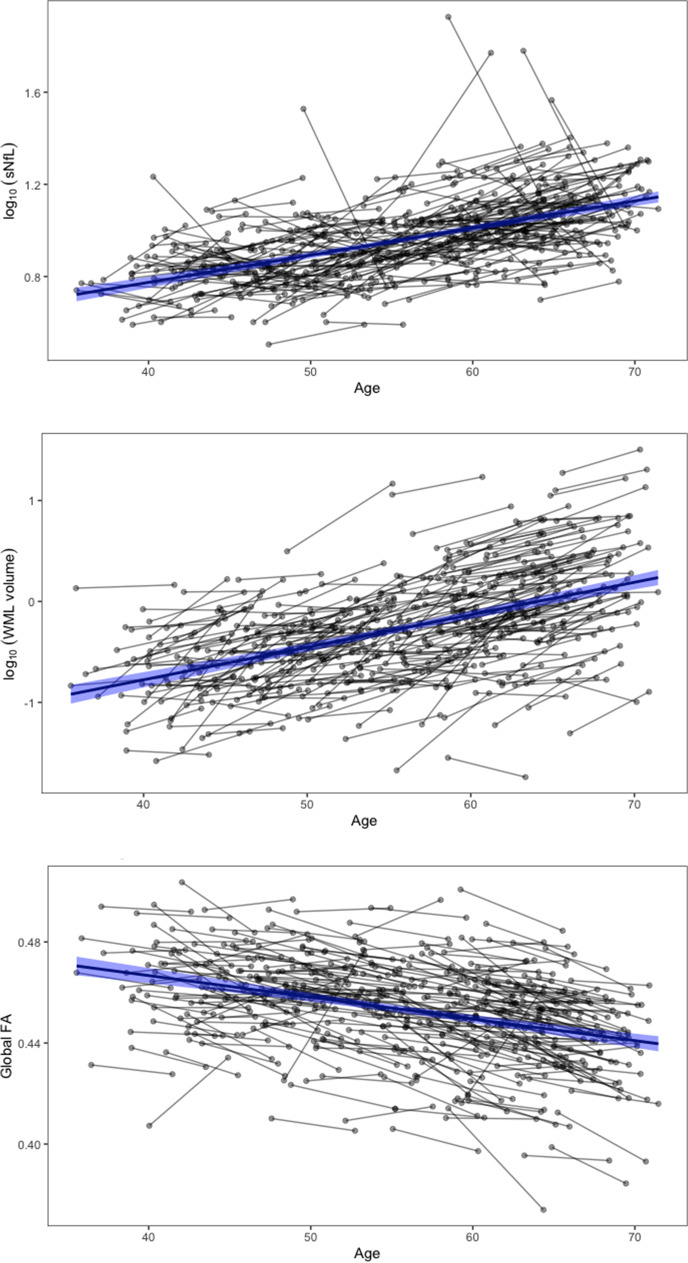
Associations between serum neurofilament light, fractional anisotropy, white matter lesion volume, and age. Gray lines correspond to the individual trajectories over time. Thick blue lines indicate the raw associations across all individual data points for illustrative purposes. The 95% confidence intervals are shown in light blue. sNfL, serum neurofilament light; FA, fractional anisotropy; WML, white matter lesions

The demographic characteristics of the sample (*n* = 307) from the general population are shown in Table 1. The mean age difference between the 2 time points was 5.39 years. Median sNfL increased by 0.9 pg/ml between the time points, while mean FA across the entire skeleton decreased by 0.006, and the median WML volume increased by 0.27 ml. The individual change in sNfL, FA, and WML volume over time is shown in Fig. 1.

**Table 1:** Demographic characteristics of the sample (*n* = 307)

	Baseline	Follow-up 2
Age (years): mean (SD)	53.08 (7.97)	58.46 (8.05)
Women: *n* (%)	153 (49.8%)	153 (49.8%)
BMI: mean (SD)	26.15 (4.07)	26.71 (4.34)
sNfL (pg/ml): median (IQR)	8.5 (4.2)	9.4 (5.25)
Log_10_ sNfL: mean (SD)	0.94 (0.18)	0.98 (0.17)
Global FA: mean (SD)	0.46 (0.02)	0.45 (0.02)
WML volume (ml): median (IQR)	0.40 (0.61)	0.67 (1.08)
Log_10_ WML volume: mean (SD)	− 0.38 (0.51)	− 0.16 (0.52)
History of stroke: *n* (%)	5 (1.6)	11 (3.6)
History of diabetes: *n* (%)	8 (2.6)	12 (3.9)
History of hypertension: *n* (%)	79 (25.7)	122 (39.7)
History of kidney disease: *n* (%)	11 (3.6)	16 (5.2)
History of cancer: *n* (%)	15 (4.9)	25 (8.1)

*SD* standard deviation, *BMI* body mass index, *sNfL* serum neurofilament light, *FA* fractional anisotropy, *WML* white matter lesion

### White matter characteristics and serum neurofilament light

Baseline global mean FA as well as log_10_ WML was significantly associated with log_10_ sNfL, respectively. Higher mean FA (*β* =  − 2.17, 95% confidence interval =  − 3.22 to − 1.13, *p* < 0.001, partial *η*^2^ = 0.05) was paralleled by lower sNfL, whereas higher WML volume (*β* = 0.11, 95% confidence interval = 0.07 to 0.15, *p* < 0.001, partial *η*^2^ = 0.10) was related to elevated sNfL. However, after adjustment for the potential confounders sex, age, and BMI, the associations with global mean FA (model 1) and log_10_ WML volume (model 2) did not reach significance as shown in Table 2.

**Table 2:** Baseline associations between sNfL, FA and WML volume

	*β* (95% CI)	*p*	Partial *η*^*2*^
Model 1			
Age	0.012 (0.010 to 0.014)	< 0.001	0.278
Female sex	− 0.035 (− 0.069 to − 0.002)	0.038	0.014
BMI	− 0.008 (− 0.013 to − 0.004)	< 0.001	0.052
FA	− 0.570 (− 1.516 to 0.375)	0.236	0.005
Model 2			
Age	0.012 (0.009 to 0.014)	< 0.001	0.234
Female sex	− 0.032 (− 0.065 to 0.001)	0.055	0.012
BMI	− 0.009 (− 0.013 to − 0.005)	< 0.001	0.056
Log_10_ WML	0.029 (− 0.008 to 0.066)	0.118	0.008

Model 1: adjusted *R*^2^ = 0.32; model 2: adjusted *R*^2^ = 0.32.

*sNfL* serum neurofilament light, *FA* fractional anisotropy, *WML* white matter lesions, *CI* confidence interval, *BMI* body mass index

**Fig. 2:**
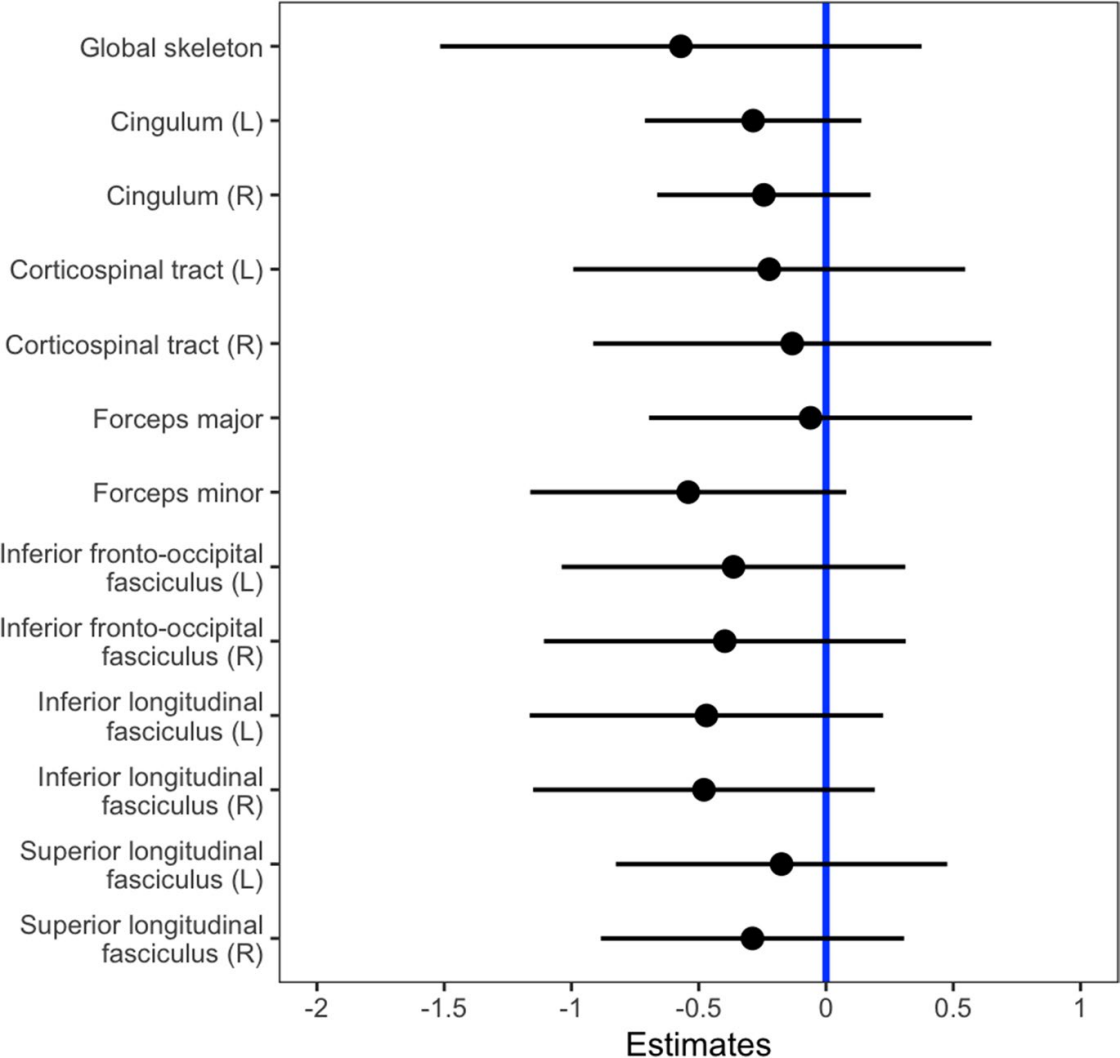
Beta estimates for tract-specific mean fractional anisotropy

**Fig. 3:**
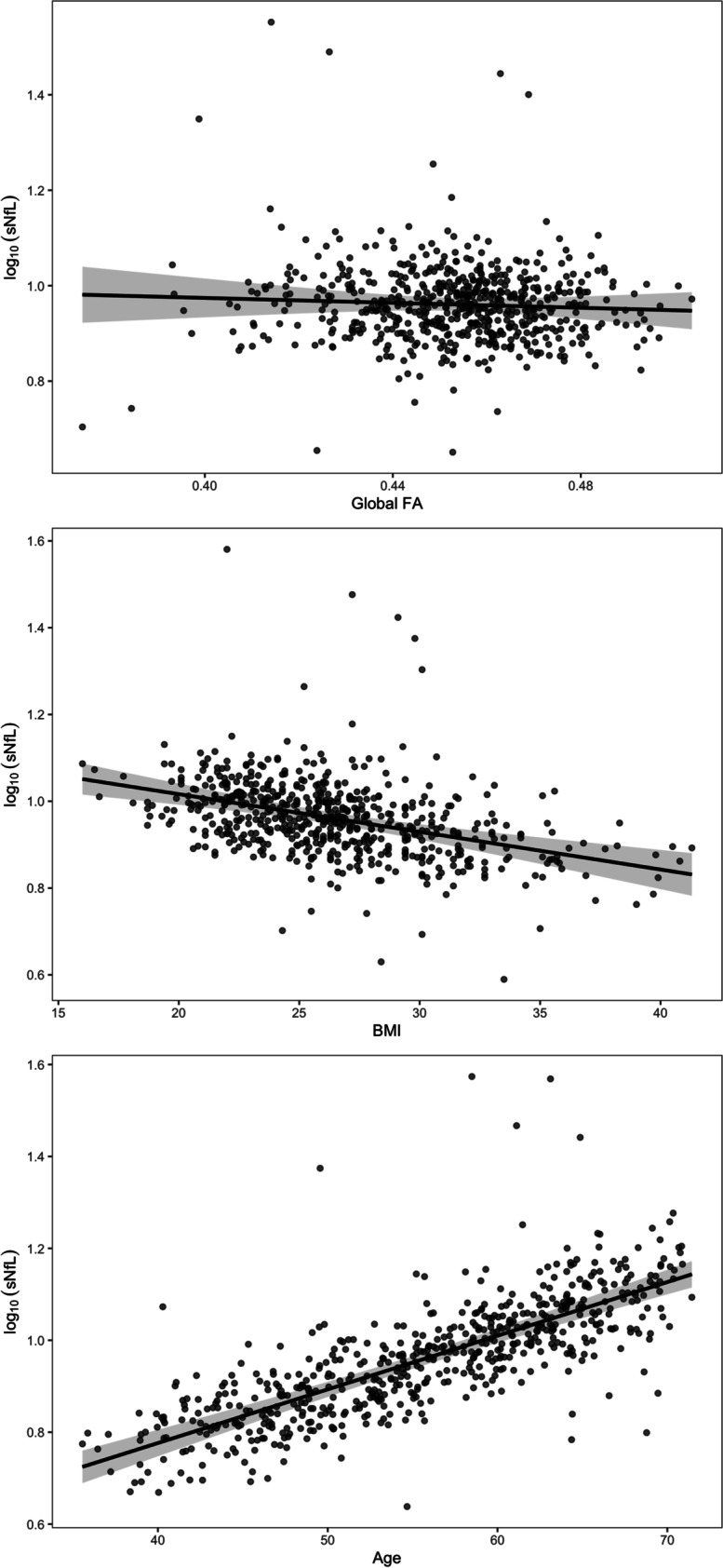
Partial residual plots showing the adjusted associations between serum neurofilament light, fractional anisotropy, age, and body mass index. Gray shades indicate the 95% confidence intervals. sNfL, serum neurofilament light; FA, fractional anisotropy; BMI, body mass index

The adjustment for age as a single confounder was sufficient to show that FA (*β* =  − 0.58, 95% confidence interval − 1.55 to 0.38, *p* = 0.23, partial *η*^2^ = 0.005) and WML (*β* = 0.02, 95% confidence interval − 0.01 to 0.06, *p* = 0.20, partial *η*^2^ = 0.005) were not significantly associated with log_10_ sNfL. Correspondingly, no tract-specific significant associations between mean FA and log_10_ sNfL were detected; tract-specific *β* coefficients are provided in Fig. 2. The longitudinal analyses of across both time points with linear mixed models did not yield significant associations between log_10_ sNfL, mean global FA, and log_10_ WML volume, respectively (Table 3). Partial residual plots showing the adjusted associations between log_10_ sNfL, mean global FA, age, and BMI are shown in Fig. 3.

**Table 3:** Longitudinal associations between sNfL, FA, and WML volume

	*β* (95% CI)	*p*
Model 1		
Age	0.012 (0.01 to 0.013)	< 0.001
Female sex	− 0.012 (− 0.039 to 0.016)	0.411
BMI	− 0.009 (− 0.012 to − 0.006)	< 0.001
FA	− 0.258 (− 0.976 to 0.459)	0.483
Model 2		
Age	0.013 (0.01 to 0.013)	< 0.001
Female sex	− 0.01 (− 0.037 to 0.017)	0.466
BMI	− 0.009 (− 0.012 to − 0.006)	< 0.001
Log_10_ WML	0.018 (− 0.01 to 0.046)	0.215

Model 1: Nakagawa’s marginal *R*^2^ = 0.35; Nakagawa’s conditional *R*^2^ = 0.67. Model 2: Nakagawa’s marginal *R*^2^ = 0.35; Nakagawa’s conditional *R*^*2*^ = 0.67

*sNfL* serum neurofilament light, *FA* fractional anisotropy, *WML* white matter lesions, *CI* confidence interval, *BMI* body mass index

## Discussion

In the present study, we aimed to explore associations between sNfL and white matter characteristics in a population-based sample with cross-sectional and longitudinal analyses. The results from the cross-sectional analyses showed that global mean FA was associated with lower sNfL, while WML volume was related to higher sNfL. However, after adjustment for potential confounders, these associations did not reach significance. These results were corroborated by tract-specific as well as the longitudinal analyses showing similar model fits and also resembling the coefficients for the confounders age and BMI. Age is a known confounder to consider [12], and BMI emerged only recently to be associated with sNfL levels [13, 36]. While it would seem plausible to expect a positive relationship between sNfL and BMI, current and previous findings show an inverse association. The etiology of this phenomenon is not completely understood, although further analyses suggest that this association may reflect differences in blood volume, body fat mass, and total body water [36, 37]. Studies or diagnostic models that aim to analyze sNfL should therefore include BMI as an easy to measure correction factor, because in some neurodegenerative disorders (e.g., Alzheimer’s disease), it has been shown that BMI declines in the prodromal stage of the disease before patients receive the diagnosis [38], which could artificially increase sNfL levels and therefore potentially confound the association with the neurodegenerative processes.

Our results suggest that white matter characteristics (FA and WML) are not related to sNfL levels beyond age in the general population. This is in line with a recent study that analyzed associations between WML volume and sNfL in the elderly population [7]. The present findings extend previous findings to a community-dwelling, middle-aged sample and also take into account potential alterations of white matter microstructure operationalized as FA. The current findings contrast with previous results from patient samples with severe neurological diseases where white matter characteristics were significantly associated with sNfL [15–17]. However, in control participants, no significant associations between sNfL and white matter characteristics were observed in contrast to patients with autosomal dominant Alzheimer’s disease [17]. Taken together with these previous findings, this strengthens the perspective that sNfL is particularly useful to identify acute cases with clinically meaningful white matter alterations. Without the need to perform a lumbar puncture, the blood-based assessment of NfL appears to provide a minimally invasive, easily implementable, and cost-efficient approach to assess the severity of neuro-axonal damage [13].

The present study is limited by the restricted follow-up period covering approximately 5 years and the baseline age span covering middle-aged adults between 35 and 65 years. We did not measure NfL levels in the cerebrospinal fluid for comparison which would have required an invasive lumbar puncture that would not be feasible in a large sample of community-dwelling participants. However, relatively high correlations between NfL levels measured in the serum, plasma, and cerebrospinal fluid have been observed in clinical samples [3]. A third limitation is that we assessed only FA as a single measure of white matter microstructure, although other measures like mean diffusivity or radial diffusivity exist. Nevertheless, none of these measures permit a conclusion about specific white matter characteristics, e.g., the degree of myelination or axonal packing density, and FA is a valid general measurement of white matter microstructure [39]. Despite a relatively high response rate of 41.5% during baseline recruitment, it cannot be ruled out that potential participants with severe diseases did not respond to an invitation by mail and were therefore not examined in the study center.

In conclusion, unadjusted FA and WML volume were associated with sNfL in a sample of middle-aged community-dwelling adults. However, after adjustment for potential confounders, neither white matter macrostructure nor microstructure was significantly associated with sNfL. The present results, based on a sample from the general population, support the perspective that changes in sNfL reflect age-related effects that also manifest in altered white matter macrostructure and microstructure. This suggests that changes in sNfL not explained by aging alone may be used to identify cases with acute neurological diseases involving white matter alterations.

## Data Availability

The underlying dataset is available upon reasonable request.
